# Seasonal differences exist in the polyunsaturated fatty acid, mineral and antioxidant content of U.S. grass-finished beef

**DOI:** 10.1371/journal.pone.0229340

**Published:** 2020-02-26

**Authors:** Raghav Jain, Sara M. Bronkema, William Yakah, Jason E. Rowntree, Chad A. Bitler, Jenifer I. Fenton

**Affiliations:** 1 Department of Food Science and Human Nutrition, Michigan State University, East Lansing, Michigan, United States of America; 2 Department of Animal Science, Michigan State University, East Lansing, Michigan, United States of America; 3 Greenacres Foundation, Cincinnati, Ohio, United States of America; The Pennsylvania State University, UNITED STATES

## Abstract

There is increased consumer interest in grass-finished beef (GFB) with retail sales reaching $272 million in 2016. GFB contains higher omega-3 fatty acid levels compared to grain-finished beef, but variations in fatty acid (FA), mineral, and antioxidant content by producers and season is poorly documented. Hence, GFB samples from cattle finished in both fall and spring were obtained from four producers representing several US sub-regions. FAs were extracted using microwave-assisted extraction, derivatized to methyl-esters, and quantified using gas chromatography-mass spectrometry. Mineral content was quantified using coupled plasma mass-spectrometry and antioxidants were quantified via UV-absorption. Overall, total omega-3 FA content was greater in beef from cattle finished in the spring (13.4 mg/100g beef) than the fall (10.3; *P*<0.001). Additionally, α-tocopherol was present in greater amounts in spring-finished beef (259 vs. 223 ug/100g beef, *P*<0.001) as was the micromineral selenium (18.2 vs. 17.3ug/100g beef, *P* = 0.008). Despite using the same feed in fall and spring, cattle from producer 4 had higher total omega-3, omega-6, and total polyunsaturated fatty acids in spring compared to fall (*P*<0.010). These results suggest there are seasonal differences in omega-3 and omega-6 fatty acids, minerals and antioxidants in grass-finished beef independent of finishing diet.

## Introduction

Over the past decade, there has been increased consumer interest in grass-finished beef (GFB), evidenced by retail sales reaching $272 million in 2016 [1]. Grass-finished beef refers to products from cattle raised solely on pasture forages without grain supplementation. Research suggests that GFB is generally higher in certain nutrients beneficial to human health and meets growing consumer preferences in meat production systems [2]. Importantly, GFB is richer in vitamins and minerals and lower in total fat than conventional grain-finished beef. Simply lowering concentrate feed in grain systems and replacing with grass feed stuffs results in lower saturated fatty acids in beef [3]. Compared to grain-finished beef, GFB also has a lower omega-6 (n-6) to omega-3 (n-3) fatty acid (FA) ratio and higher amounts of the vitamin A precursor β-carotene [4]. These observations have supported the growing shift from grain-finished beef to GFB. Interestingly, there is no USDA definition of the ‘grass-fed’ label in regards to production strategies or nutritional profile, making it difficult for consumers to evaluate healthfulness when comparing different GFB products [5]. The standard reference for GFB on the USDA food database (NDB Id: 13047) does not include information on individual FA breakdown or data on nutrient variation, both of which are crucial to gaining a deeper understanding of the benefits of GFB [6]. Further, beef products carrying the ‘grass-fed’ label originate from cattle from a variety of landscapes, climates and agricultural systems, and the interaction of these factors and their effects on the nutritional density of GFB requires more investigation.

Previous research from our group demonstrated that GFB nutritional content varies significantly by producer/production system [7]. Variations in mean total n-6 and n-3 FAs contributed to a 20-fold range in observed n-6 to n-3 ratios (1.80 to 28.3) with some reported ratios much higher than previously suggested for GFB. There is significant consumer interest in reducing n-6 FA consumption due to their purported pro-inflammatory properties linked to risk of rheumatoid arthritis, obesity, and cardiovascular disease, among others [8]. In contrast, omega-3 FAs are widely recognized for their role in reducing the risk of cardiovascular disease and diabetes, among other benefits [9] and red meat can be a source of n-3 FAs depending on feed systems [10, 11]. Hence, it is important to investigate the high variation in the n-3 and n-6 FA content of GFB to understand factors that influence FA levels such as season. For example, cattle finished in spring rather than fall have higher n-3 FAs [12]. Finishing in spring also increases the saturated FA (SFA) stearic acid, and the monounsaturated FA (MUFA) oleic acid in beef tissue [12, 13]. Stearic acid has a net neutral effect on serum cholesterol unlike the SFAs palmitic and myristic acid, which are associated with increases in serum LDL cholesterol [14]. Increases in LDL levels are associated with a higher risk of coronary events and stroke [15]. Oleic acid has beneficial effects against cancer and other inflammatory diseases [16]. It is unclear the exact mechanisms through which season affects GFB nutrient density and quality, though it may be related to changes in the forages cattle graze due to seasonal climate and plant composition changes.

The phytochemical and overall biochemical richness of pastures determines the nutrients available for consumption by cattle and affects the nutritional profile of the beef [17]. For example, simply allowing cattle to graze on fresh grass rather than harvested silage results in higher oleic acid, linoleic acid (LA), and alpha-linolenic acid (ALA) content deposition in the muscle tissue, presumably due to higher lipid oxidation in harvested feed [18]. Types of grass and forage species affect available nutrients for consumption as well. Orchard grass, tall fescue and perennial ryegrass are all richer in the n-3 ALA than alfalfa, which has approximately 2 mg more of the n-6 LA per gram than the other grasses [19]. Additionally, adding certain forages such as red clover to silage can boost LA and ALA levels in beef tissue above levels present if cattle graze only on fresh grass [20]. Compounds in red clover reduce rumen FA biohydrogenation, preventing the metabolism of n-3 FAs as well as conjugated linoleic acid (CLA) [21, 22]. CLA is group of dietary polyunsaturated FAs (PUFA) that are isomers of LA. The major form of CLA produced in both humans and ruminants is the 9Z, 11E CLA isomer (also known as rumenic acid), and its consumption is linked to reductions in obesity, cancer and diabetes [23–25]. The nutrient composition of pasture grass and forages is dependent on numerous factors including soil mineral content, soil moisture, weather, and climate, as well as plant species, maturity, and leaf to stem ratio [26, 27]. Seasonal changes affect available plants for consumption and despite the ability of animals to adapt to changes in forages, the marked decrease in overall pasture forage mass during the end of growing season cannot be fully compensated for by cattle [28]. Therefore, in temperate climates present throughout the USA, seasonal variation in forages likely affects GFB nutrient composition, causing both inter- and intra-producer GFB nutrient variation throughout the year.

The objective of this study was to compare seasonal variations in mineral, antioxidant and fatty acid content of GFB. These results provide crucial insights into the impact of season and feed on the nutritional composition of GFB products among various producers across the country and may be utilized for producer recommendations to enhance the nutrient density of the GFB product.

## Materials and methods

This project received Exempt 2 status from the Michigan State University Institutional Review Board, IRB# x16-1273e, October 12^**th**^, 2016.

### Sample collection

The samples analyzed in this study were cut from the anterior portion of the strip loin (IMPS/NAMP 180 Beef Loin, Strip Loin). Twelve producers submitted samples for nutrient analysis, as previously described [7]. Samples harvested in the fall were collected between September 2016 –February 2017 and spring harvested samples were collected between June 2017—August 2017. Four producers submitted at least 25 samples from cattle finished in both fall and spring and were the focus of the present study. Producer names and locations cannot be specified due to Institutional Review Board specified survey confidentiality, however, the samples originated from Nebraska, Oklahoma, and Georgia. Samples spent approximately 31 days in a freezer by producers prior to submission and were stored at -80°C prior to analysis.

### Beef nutrient analysis

All solvents were analytical grade and purchased from Sigma-Aldrich (St. Louis, Missouri, USA). Beef amples were analyzed for mineral, antioxidant and fatty acid content as detailed previously [7]. Briefly, a microwave assisted extraction method was used to extract FAs from 400 mg of beef samples by heating samples at 55°C for 15 mins in 8 mL of a 4:1 (v/v) solution of ethyl acetate: methanol containing 0.1% (w/v) butylated hydroxytoluene. A CEM Corporation (Matthews, North Carolina, USA) Mars 6 microwave digestion machine was used for FA extraction. Fatty acids were derivatized to FA methyl-esters (FAMEs) by heating for 1.5h in a 1.09M methanolic HCl solution. Samples were analyzed using the Perkin-Elmer (Waltham, MA, USA) 680/600S GC-MS equipped with an Agilent Technologies (Santa Clara, CA, USA) DB-23, 30-m column for FAME quantification. GC profile was as follows: initial temperature at 100°C for 0.5 min; ramp 7.0°C/min to 245°C; hold 2 min. GC-MS data was analyzed with MassLynx V4.1 (Waters Corporation; Milford, MA, USA). For mineral analysis, dried beef samples were digested overnight at 75°C in 2 mL of nitric acid and minerals were quantified using an Agilent 7900 inductively coupled plasma-mass spectrometer. For antioxidant analysis, beef samples were homogenized and frozen in water prior to the extraction of β-carotene and α-tocopherol with hexane. The antioxidants were separated using a Waters 2 Acquity System (Waters Corporation, Milford, MA) and quantified using UV-spectroscopy.

### Statistical analysis

Fatty acids were reported as mg FA/100g of tissue and percent of total of FAs. Macrominerals were expressed as mg mineral/100g tissue, while microminerals and antioxidants are expressed in μg mineral/100g tissue. Mean ± standard error of the mean (SEM) were presented for FAs, antioxidants and minerals. Parametric analyses were used for antioxidants, minerals and FAs. All variables were log_10_ transformed prior to significance testing to ensure normality due to skewness in some variables. The Welch t-test was used to assess intra-producer FA differences as the assumption of equal sample sizes is not required. Seasonal differences in total FAs were determined by controlling for producer associated differences using a two-way ANOVA with interaction: log_10_(fatty acid/mineral/antioxidant) = season + producer + season*producer. Differences in specific nutrients by producer were further evaluated using a one-way ANOVA and Tukey’s post-hoc analysis. Data analysis was performed using R (R version 3.4.3, Vienna, Austria). *P*-values ≤0.05 were considered statistically significant. The dataset and R code have been made available in the supplement (S1 and S2 Appendix, respectively).

## Results

Producer comparisons of beef cattle by region, finishing diets and age at harvest are summarized in Table 1. Producers are related to a previous study by Bronkema et al. and the producer numbers match those in that study [7]. The cattle age at harvest for all four producers ranged between 23 and 28 months. A total of 512 samples were analyzed, approximately half of which were from Producer 8 (n = 250). In total, 202 samples were collected in the fall and 310 during spring. The type of cattle were Angus or Angus cross for all producers. Finishing diets were fed beginning 60 days prior to slaughter, as reported by producers. Fall and spring finishing diets varied for producers 2 and 8 but not 4, and producer 5 did not disclose feeding information.

**Table 1:** Producer comparison of cattle age and finishing diet^1^.

Producer	Total samples	Region	Fall finishing diet	Spring finishing diet	Age at harvest (months)
2	n = 75	Midwest	Brown midrib (BMR) forage sorghum, oat/pea/triticale silage, apple cider vinegar, cane molasses, soybean hulls	Oat/pea silage, alfalfa, BMR silage, cane molasses, soybean hulls	23–26
	F = 25				
	S = 50				
4	n = 106	Southeast	Summer annuals and warm season perennial pasture with either cool season baleage or cool season annuals and warm season annual baleage	Same as fall	23
	F = 35				
	S = 71				
5	n = 81	Midwest	NA	NA	NA
	F = 43				
	S = 38				
8	n = 250	Southwest	Seasonal forages	Winter annuals (barley, wheat) and sorghum sudan silage or native pasture and BMR sudan	24–28
	F = 99				
	S = 151				

^1^Finishing diet fed beginning 60 d prior to slaughter as reported by producers. Data not reported is indicated by NA. F = fall, S = spring.

Total FA (mg FA/100g tissue) of beef significantly varied by producer (Table 2) in fall and spring (*P*<0.001). Samples from producer 4 had the highest overall FA content (1122±54.0), followed by producers 2, 5 and 8. Producer 2 had significantly higher total FA in the fall (1250±123) compared to spring (778±54.2; *P* = 0.001), while producer 4 had higher total FA in the spring (*P*<0.001). No significant seasonal differences in total FAs was found for producers 5 and 8.

**Table 2:** Seasonal variation in total fatty acid levels in mg FA/100g tissue by producer (mean ± SEM).

	Producer				
	2	4	5	8	P-value^1^
Total FA	935±60.1^b^	1122±54.0^a^	835±59.4^b^	544±17.2^c^	**<0.001**
Fall (F)	1250±123^a^	823±68.4^b^	828±83.7^b^	539±27.0^c^	**<0.001**
Spring (S)	778±54.2^b^	1269±66.9^a^	842±85.2^b^	548±22.3^c^	**<0.001**
F vs S p-value^2^	**0.002**	**<0.001**	0.828	0.904	

^1^One-way ANOVA was used to compare overall fatty acid differences across producers and Tukey’s post-hoc test for multiple comparisons was performed. Means in a row without a common letter differ, *P*<0.05.

^2^Welch-approximation t-test was performed to compare seasonal differences in total fatty acids within producers.

Seasonal variation of specific fatty acids reported in mg FA/100g tissue are shown in Table 3, and by percent of total FA in S1 Table. After controlling for producer variation, total SFAs and MUFAs were higher in spring compared to fall, although not statistically significant. The n-6 arachidonic acid (AA) and dihomo-gamma linolenic acid (DGLA) were significantly higher in the spring compared to fall (*P*<0.010). Similarly, the n-3 FAs ALA, eicosapentaenoic acid (EPA), docosapentaenoic acid (DPA) and docosahexaenoic (DHA) were significantly higher in spring compared to fall (*P*<0.001). Consequently, the n-6:n-3 ratio was significantly lower in spring compared to fall (*P*<0.001).

**Table 3:** Overall, fall and spring mean ± SEM data beef fatty acids (mg FA/100g tissue) of all producer data combined.

Fatty acid	Carbon #	Overall (n = 512)	Fall (n = 202)	Spring (n = 310)	P-value^1^
Myristic	14:0	16.2±0.63	15.5±0.94	16.6±0.85	0.735
Palmitic	16:0	216±6.65	211±10.7	219±8.49	0.451
Margaric	17:0	8.43±0.31	7.79±0.40	8.84±0.43	0.109
Stearic	18:0	99.5±2.59	96.6±4.17	101±3.30	0.227
Tricosanoic	23:0	0.32±0.01	0.27±0.01	0.35±0.01	**<0.001**
Lignoceric	24:0	0.55±0.01	0.41±0.01	0.60±0.01	**<0.001**
Total SFA		340±10.0	331±16.0	346±12.9	0.355
Myristoleic	14:1n-7	4.45±0.18	4.36±0.23	4.51±0.25	0.098
Palmitoleic	16:1n-7	25.1±0.87	23.4±1.24	26.1±1.19	0.175
Oleic	18:1n-9	311±9.79	299±15.4	319±12.7	0.226
Total MUFA		341±10.8	327±16.8	350±14.0	0.229
Linoleic	18:2n-6	50.3±1.20	47.5±1.53	52.2±1.71	0.100
DGLA	20:3n-6	3.87±0.08	3.38±0.10	4.19±0.11	**<0.001**
Arachidonic	20:4n-6	17.8±0.32	16.9±0.45	18.5±0.43	**0.003**
Total n-6		72.0±1.54	67.7±1.98	74.8±2.18	**0.018**
ALA	18:3n-3	5.16±0.16	4.70±0.24	5.45±0.21	**<0.001**
EPA	20:5n-3	3.14±0.10	2.58±0.13	3.49±0.13	**<0.001**
DPA	22:5n-3	3.83±0.08	3.01±0.10	4.36±0.11	**<0.001**
DHA	22:6n-3	0.32±0.01	0.28±0.01	0.34±0.01	**<0.001**
Total n-3		12.2±0.33	10.3±0.45	13.4±0.44	**<0.001**
9Z, 11E CLA	18:2n-7	1.55±0.06	1.28±0.08	1.73±0.08	**<0.001**
Total PUFA		85.8±1.43	79.3±1.83	90.0±2.01	**<0.001**
n-6:n-3		11.7±0.61	13.5±1.05	10.5±0.72	**<0.001**
SFA:PUFA		3.93±0.09	4.17±0.17	3.78±0.10	**0.004**

^1^P-value of seasonal term from two-way ANOVA of pooled data with model: FA = season + producer + producer*season. ALA, alpha-linolenic acid; CLA, conjugated linoleic acid; DGLA, dihomo-gamma linolenic acid; DHA, docosahexaenoic acid; DPA, docosapentaenoic acid; EPA, eicosapentaenoic acid; FA, fatty acid; MUFA, monounsaturated FA; n-3, omega-3; n-6, omega-6; n-6:n-3 ratio, total omega-6/total omega-3 ratio; PUFA, polyunsaturated fatty acid; SFA, saturated FA. SFA:PUFA = Total SFA/Total PUFA

Table 4 shows mineral and antioxidant content in beef tissue. Sodium, phosphorus and β-carotene were significantly higher in the fall compared to spring. However, the macrominerals magnesium and potassium, microminerals iron, zinc, and selenium, and α-tocopherol were significantly higher in the spring.

**Table 4:** Mineral and antioxidant concentration of beef tissue (mean ± SEM).

Macromineral (mg/100g)	Overall (n = 512)	Fall (n = 202)	Spring (n = 310)	P-value^1^
Sodium	40.8±0.29	42.5±0.43	39.7±0.38	**<0.001**
Magnesium	25.8±0.07	25.5±0.12	26.1±0.07	**<0.001**
Phosphorus	209±0.49	215±0.88	205±0.45	**<0.001**
Sulfur	206±0.56	206±0.91	205±0.71	0.292
Potassium	423±1.00	419±1.85	425±1.11	**<0.001**
**Micromineral (μg/100g)**				
Iron	2125±47.7	2122±115	2127±23.8	**0.012**
Zinc	4085±32.7	3976±45.5	4155±44.7	**0.009**
Copper	63.3±0.69	64.0±0.97	62.9±0.94	0.391
Selenium	17.8±0.26	17.3±0.45	18.2±0.32	**0.008**
Molybdenum	2.76±0.12	2.59±0.20	3.11±0.12	0.269
**Antioxidant (μg/100g)**				
β-carotene	31.9±0.45	33.6±0.75	30.3±0.55	**0.030**
α-tocopherol	245±5.73	223±10.7	259±6.25	**<0.001**

^1^Statistical significance of seasonal term from two-way ANOVA of pooled data with model: mineral/antioxidant = season + producer + producer*season.

To investigate which producers contributed to significant seasonal differences in FAs, n-3 and n-6 fatty acids were further categorized by season and producer as shown in Table 5 (data for all FAs available in S2 Table). Total n-3 FAs were significantly higher in the spring compared to fall for 3 out of 4 producers. Producers 2 and 8 had higher total n-3 FAs compared to 4 and 5, but lower total n-6 FAs regardless of season. Cattle from producer 4 had significantly higher total n-3 and n-6 FAs in the spring compared to fall (*P*<0.05). Macro/micro mineral content (Table 6) varied between seasons with no clear trend. Detection of β-carotene varied from producers, ranging between 6.7% of samples in producer 2 to 72.4% in producer 8. Samples from producer 4 had the highest α-tocopherol levels in the fall amongst all producers, which was also significantly higher compared to its levels in the spring.

**Table 5:** Polyunsaturated fatty acid (mg FA/100g tissue) differences by producer and season (mean ± SEM)^1^.

Fatty acid		Producer 2 (n = 75)	Producer 4 (n = 106)	Producer 5 (n = 81)	Producer 8 (n = 250)
ALA					
	Fall	6.84±0.59^a^	1.46±0.12^b*^	2.00±0.30^b^	6.50±0.29^a^
	Spring	5.83±0.26^a^	3.26±0.47^b^	1.76±0.16^c^	7.28±0.27^a^
EPA					
	Fall	2.90±0.36^a^	0.76±0.07^b*^	1.30±0.16^b*^	3.60±0.16^a*^
	Spring	3.00±0.20^b^	1.52±0.20^d^	1.79±0.14^c^	4.87±0.18^a^
DPA					
	Fall	3.94±0.24^a*^	1.59±0.11^b*^	1.99±0.15^b*^	3.72±0.13^a*^
	Spring	4.67±0.20^a^	2.79±0.20^b^	2.88±0.17^b^	5.36±0.14^a^
DHA					
	Fall	0.28±0.03^a*^	0.19±0.01^a^	0.23±0.02^a*^	0.30±0.02^a*^
	Spring	0.35±0.02^a^	0.23±0.01^b^	0.30±0.02^ab^	0.37±0.01^a^
Total n-3					
	Fall	14.0±1.08^a^	3.76±0.26^b*^	5.30±0.56^b*^	13.9±0.54^a*^
	Spring	13.8±0.59^b^	7.47±0.85^c^	6.55±0.39^c^	17.8±0.54^a^
Linoleic					
	Fall	43.4±3.02^c^	70.3±3.26^a*^	59.2±3.29^b^	35.4±1.38^c^
	Spring	36.6±1.41^c^	91.3±3.52^a^	64.1±3.47^b^	35.9±1.14^c^
DGLA					
	Fall	3.38±0.24^bc^	4.55±0.24^a*^	3.88±0.22^ab*^	2.75±0.11^c*^
	Spring	3.41±0.13^c^	6.49±0.23^a^	4.64±0.20^b^	3.25±0.09^c^
Arachidonic					
	Fall	14.2±0.70^b^	22.8±1.46^a*^	18.8±0.89^a^	14.6±0.44^b^
	Spring	15.0±0.54^c^	27.5±0.98^a^	18.9±0.89^b^	15.3±0.39^c^
Total n-6					
	Fall	61.0±3.76^c^	97.6±4.58^a*^	81.8±4.11^b^	52.7±1.80^c^
	Spring	55.1±1.84^c^	125±4.52^a^	87.6±4.20^b^	54.5±1.53^c^
9Z, 11E CLA					
	Fall	1.66±0.23^a^	0.83±0.13^c*^	1.21±0.22^bc^	1.37±0.11^ab*^
	Spring	1.18±0.14^b^	1.85±0.19^a^	1.26±0.22^b^	1.98±0.12^a^
Total PUFA					
	Fall	76.6±4.35^bc^	102±4.75^a*^	88.3±4.07^ab^	68.0±1.84^c*^
	Spring	70.0±2.35^c^	135±4.22^a^	95.4±4.54^b^	74.2±1.56^c^
n-6:n-3 ratio					
	Fall	4.78±0.35^c^	30.1±2.29^a*^	23.4±2.34^b^	5.62±0.83^c*^
	Spring	4.10±0.10^c^	27.1±1.91^a^	14.9±1.02^b^	3.75±0.22^d^

^1^One-way ANOVA with Tukey’s post-hoc analysis was performed to compare of FA data between producers in each season. Mean values in a given row without a common letter differ, *P*<0.05. Asterisk (*) indicates significantly different in fall compared to spring within producer by Welch t-test, *P*<0.05. ALA, alpha-linolenic acid; CLA, conjugated linoleic acid; DGLA, dihomo-gamma linolenic acid; DHA, docosahexaenoic acid; DPA, docosapentaenoic acid; EPA, eicosapentaenoic acid; n-6:n-3 ratio, total omega-6/total omega-3 FA ratio; PUFA, polyunsaturated fatty acid.

**Table 6:** Macromineral (mg/100g tissue), micromineral (ug/100g tissue) and antioxidant (ug/100g tissue) comparison by producer and season (mean ± SEM)^1^.

Minerals and Antioxidants		Producer 2 (n = 75)	Producer 4 (n = 106)	Producer 5 (n = 81)	Producer 8 (n = 250)
Sodium					
	Fall	46.0±0.80^a*^	44.0±0.80^ab*^	41.7±0.83^b^	41.4±0.69^b*^
	Spring	36.7±0.63^c^	39.9±0.66^ab^	43.0±1.08^a^	39.8±0.60^b^
Magnesium					
	Fall	24.0±0.31^b*^	25.4±0.18^a*^	25.7±0.28^a*^	25.7±0.18^a^
	Spring	25.9±0.13^b^	25.9±0.14^b^	26.6±0.23^a^	26.1±0.10^ab^
Phosphorus					
	Fall	204±1.93^b^	207±1.57^b*^	219±1.93^a*^	217.9±1.15^a*^
	Spring	202±0.84^c^	203±0.80^bc^	210±1.51^a^	206±0.65^b^
Sulphur					
	Fall	207±2.29^ab*^	199±1.46^b*^	206±1.77^ab^	209±1.44^a*^
	Spring	201±1.46^b^	210±1.44^a^	211±1.99^a^	203±1.02^b^
Potassium					
	Fall	395±5.07^c*^	401±2.86^c*^	418±3.20^b^	432±2.33^a^
	Spring	414±2.27^b^	428±2.12^a^	417±3.23^b^	430±1.57^a^
Iron					
	Fall	1994±73.8^a^	2118±239^a^	2524±498^a^	1982±36.4^a*^
	Spring	2139±55.4^a^	2112±53.6^a^	2086±72.7^a^	2141±33.1^a^
Zinc					
	Fall	3831±109^a*^	3849±121^a*^	4035±108^a^	4033±61.7^a^
	Spring	3553±89.5^c^	4631±101^a^	4250±116^ab^	4109±54.5^b^
Copper					
	Fall	60.3±1.23^ab^	71.6±3.70^a^	68.8±2.15^a^	59.2±0.59^b^
	Spring	61.1±1.08^ab^	62.6±1.22^ab^	69.2±1.73^a^	62.1±1.74^b^
Selenium					
	Fall	21.0±1.25^a^	12.9±0.56^c*^	22.5±1.18^a*^	15.6±0.45^b^
	Spring	23.3±0.40^a^	16.1±0.54^b^	25.1±0.69^a^	15.7±0.34^b^
α-tocopherol					
	Fall	142±22.3^b^	368±10.4^a*^	185±17.5^b^	208±16.8^b*^
	Spring	142±5.87^b^	290±11.8^a^	171±10.4^b^	305±7.98^a^
β-carotene^2^					
	% detected	6.7%	19.8%	7.4%	72.4%
	Fall	37.4±1.64^a^	26.4±0.54^a^	30.3±1.23^a^	34.4±1.12^a*^
	Spring	<LOD	32.3±2.81	<LOD	30.1±0.56

^1^One-way ANOVA and Tukey’s post-hoc analysis was performed to compare mineral/antioxidant values between producers in each season. Mean values in a given row without a common letter differ, *P*<0.05. Asterisk (*) indicates significantly different in fall compared to spring within producer by Welch t-test, *P*<0.05.

^2^β-carotene measurements below limit of detection (LOD) were excluded from the analysis. Percent of samples in which β-carotene was detected is provided in the table.

## Discussion

The potential healthfulness of GFB is one of the primary drivers of consumer interest in the product [29]. Research on GFB has mainly focused on its low ratio of n-6 to n-3 FAs [4, 7, 13, 22]. A 2:1 n-6 to n-3 dietary ratio is considered ideal [30], however, typical ‘western diets’ are estimated to be 20:1 and grain-finished beef have reported ratios up to 13.6:1 [31, 32]. In this study, the n-6 to n-3 ratio was lower in spring samples but slightly higher than previous reports on GFB [32–34]. Total n-6 FAs were significantly higher in spring samples, specifically the FAs AA and DGLA. While AA is considered pro-inflammatory, DGLA is an n-6 FA that can help prevent inflammation, a hallmark of diseases such as obesity, diabetes, and cancer, by blocking the production of pro-inflammatory lipids such as prostaglandins [35, 36]. More notably, the reduction of the ratio in spring was driven by an increase of 3 mg/100g tissue of total n-3 FAs which is considered more important in improving health outcomes than simply a reduction in n-6 FAs [37]. The increase of n-3 FAs in spring GFB is likely due not only to higher total n-3 in pasture forages, but also higher antioxidant content in spring than fall. Antioxidants reduce lipid oxidation and help prevent rumen biohydrogenation of certain FAs such as the n-3s [20, 38, 39]. This also extends the shelf-life of GFB [40]. Two important lipid soluble antioxidants are β-carotene and α-tocopherol. β-carotene values ranging from 16–74 ug/100g tissue have been reported in GFB [38, 41], with both α-tocopherol and β-carotene levels supposedly higher in spring and summer samples than fall due to increased pasture grass intake [42]. In this study, β-carotene was significantly higher in the fall though the actual values were similar in both spring and fall. Importantly, α-tocopherol, approximately seven times more abundant than β-carotene regardless of season, was significantly higher in spring samples and, therefore, may have contributed more heavily to preserving n-3 FAs than β-carotene. Another important antioxidant quantified in this study was the micromineral selenium. Selenium plays an important role in human metabolism and deficiencies can increase the risk of heart disease and even cancer [43]. Spring GFB had a higher Se content than fall, and Se may also contribute to preserving n-3 FAs in tissue by preventing lipid peroxidation [44]. Strategies aiming to improve the n-3 content and n-6:n-3 ratio of fall GFB should therefore focus not only on increasing the n-3 content of forages, but also total antioxidant content.

While seasonal trends in FA content were clear in the overall sample set, some variation was evident from producer to producer. Several studies have shown that variations in forage species in the finishing diet can affect the FA profile of GFB. For example, GFB from cattle finished on alfalfa had lower levels of stearic acid than those finished on bermudagrass, cowpea, chicory or pearl millet [45]. In a separate study, beef from cattle finished on alfalfa showed a greater amount of ALA than those finished on mixed pasture or pearl millet [46]. Additionally, cattle finished on birdsfoot trefoil, a perennial legume, have increased ALA and DPA compared to that finished solely on grass [2]. Because of the survey-based nature of this study, information on diets fed to the cattle is limited, and based only on self-reported data from the producer. Producer 2 indicated the most variation in diets between season, with a difference in the fresh forage base (forage sorghum in fall vs alfalfa in spring), as well as some variation in the harvested feeds supplemented in each season. Despite this variation, the total n-3 and total n-6 FA content of the beef was not different between seasons. Producer 8 also indicated differences between seasonal diets, with the addition of harvested feeds in the spring diet of some of their cattle. Despite the addition of harvested feeds to the diet, which has been reported to reduce n-3 FA content of beef compared to solely fresh forages [3], total n-3 FA were greater in beef from cattle finished in the spring, and the n-6:n-3 ratio was significantly lower. Producer 4 did not indicate specific differences in the diets fed to cattle between seasons, however, both n-3 and n-6 FA were significantly higher in the spring samples. Therefore, while differences in feed may explain some of the variation in GFB FA profiles, there are likely seasonal factors that affect the FA profile of GFB going beyond simply the finishing diets.

Overall, mineral values were similar to those previously reported in GFB [32]. It has been suggested that the mineral content of GFB is highly dependent on the soil mineral composition on which forages are grown [47], though analysis of pasture soil and grass mineral content has shown little correlation [48]. However, seasonal trends for plant species, not soil, were found, with higher plant mineral content during the spring months than late summer/fall [49]. Unfortunately, information on plant species of the feeds in this study is limited. Still, despite producers being from different regions of the USA and employing different feeds for cattle, the mineral content of both fall and spring samples was fairly consistent and there was no clear relationship between overall mineral content and season. There is a growing amount of evidence suggesting beef mineral content is largely affected by genetics and muscle [50]. In the present study, producers reported similar breeds of cattle, Angus or Angus-cross, and the same strip loin cut was taken from all animals for analysis, perhaps an explanation for the similarities in mineral contents across producers.

In addition to feed, geographical region and the related climate are known to affect cattle growth, weight and, hence, FA content [51, 52]. Therefore, while seasonal variations in weather and feed may help explain the changes in relative amounts of FA within producers and in different seasons, large differences in inter-producer FA content may also be dependent on the overall climate in the regions from which samples originated. For example, the total FA content of Producers 2, 4 and 5 ranged from 705–969 mg FA/100g tissue whereas Producer 8 was significantly lower at 485 mg FA/100g. Producer 8 is located in the southern plains of the USA which has experienced increased occurrences of consistently hot weather punctuated with short bouts of extreme weather rather than prolonged, differing seasons [53]. The effects of long term heat stress on the FA profile of cattle is poorly studied, though there is some evidence to suggest that adipocytes are conserved during hyperthermia and skeletal muscle breakdown provides nutrients to aid in survival of cattle during such stress [54]. It is possible that cattle from producers 2, 4 and 5, located in different climates, may have experienced fewer or less consistent heat stress events than cattle from producer 8, potentially affecting the fat content of muscle tissue. However, much more research is needed on how long-term heat stress affects the growth and nutrient composition of the animals before any conclusions can be made. This is particularly important for GFB, as cattle roam freely in pastures in full exposure to the weather and high temperatures may adversely affect grazing patterns.

This study has limitations and strengths. As the study relied on self-reported producer data, some information regarding carcass drying time and feed composition was either incomplete or missing entirely, and both factors can affect the GFB nutritional profiles. Due to study parameters and restrictions, analysis of pasture grass and forage nutrient content was not possible, making it difficult to determine the relationships between nutrients in feed and subsequent absorption into muscle tissue. Additionally, the precise location of producers cannot be revealed due to survey confidentiality, limiting analysis on the effects of geographical and weather/climate factors on the nutrient content of beef samples. This study has numerous strengths as well. This is one the few studies that analyzed GFB originating from the USA from multiple producers across diverse regions for fatty acid, mineral, and antioxidant content. Additionally, a large sample size of GFB samples was employed to make meaningful comparisons of the quantified nutritional content of beef samples available throughout the year. This study collected samples marketed to consumers, providing direct examples of GFB products available in the market. Hence, the information provided here sheds light on the large variation in GFB products from the same producer, among different producers, and between seasons, highlighting the need for better systems to define and clarify the nutritional content of GFB products sold to consumers. Future studies should focus on the nutritional content of GFB products from different producers located within the same region (i.e. Midwest) to determine if and to what extent locality affects the nutritional consistency of GFB products. More studies are also needed on the impact of specific forages on the nutritional profile of GFB, as this information may help design feeding strategies to improve and maintain the nutritional content of beef throughout the year. Finally, more work needs to be done to create classifications for GFB products that allow consumers to compare different products and better understand the nutritional consequences of their food.

## Conclusions

This was an in-depth analysis of the seasonal variation in the antioxidants, minerals, and FAs of GFB samples collected from four producers located throughout the USA. Overall, total n-3 FAs were higher in spring (13.4 mg/100g beef) samples than fall (10.3; P<0.001), as was the antioxidant α-tocopherol and several microminerals including selenium. Significant nutritional differences were observed in fall and spring samples from producers reporting the same feeding strategy in both seasons, hence finishing diets may not solely explain seasonal nutritional variability in GFB. Factors such as local climates and changes in pasture composition between seasons were beyond the scope of this study but likely affected the nutrient deposition in GFB tissue. Future studies should analyze whether relative nutritional differences in forages are related to changes observed in GFB nutrients. Additionally, future studies may explore the effects of long-term heat stress on the nutritional content of GFB as that may explain some of the nutritional variation observed between producers from different regions of the USA. Still, the large sample size and wide scope of this study highlights the clear need for a better classification system to identify and distinguish among GFB products of different origins and may be used to inform future studies and interventions aimed at improving the nutrient quality of available GFB.

## Supporting information

S1 Appendix The dataset used for analysis. (XLSX)

S2 Appendix R code. The R code used for statistical analysis as an .html file.
(HTML)

S1 Table Overall, fall and spring median ± IQR data of analyzed fatty acids (% total FA). ALA, alpha-linolenic acid; CLA, conjugated linoleic acid; DGLA, dihomo-gamma linolenic acid; DHA, docosahexaenoic acid; DPA, docosapentaenoic acid; EPA, eicosapentaenoic acid; FA, fatty acid; MUFA, monounsaturated FA; n-3, omega-3; n-6, omega-6; PUFA, polyunsaturated fatty acid; SFA, saturated FA
(XLSX)

S2 Table Full FA breakdown by producer and season. Welch t-test used to asses if significant differences existed between seasons for a given producer. One-Way ANOVA used to compare seasonal FAs among producers with Tukey’s post-hoc test. Results of Tukey’s test are in separate tabs for each season. ALA, alpha-linolenic acid; CLA, conjugated linoleic acid; DGLA, dihomo-gamma linolenic acid; DHA, docosahexaenoic acid; DPA, docosapentaenoic acid; EPA, eicosapentaenoic acid; FA, fatty acid; MUFA, monounsaturated FA; n-3, omega-3; n-6, omega-6; PUFA, polyunsaturated fatty acid; SFA, saturated FA.
(XLSX)

## References

[1] Cheung R, McMahon P, Norell E, Kissel R, Benz D. Back to grass: the market potential for grassfed beef. New York: Stone Barns Center For Food and Agriculture; 2017.

[2] Chail A, Legako JF, Pitcher LR, Griggs TC, Ward RE, Martini S, et al Legume finishing provides beef with positive human dietary fatty acid ratios and consumer preference comparable with grain-finished beef. J Anim Sci. 2016;94(5):2184–97. 10.2527/jas.2015-0241 27285714

[3] French P, Stanton C, Lawless F, O'Riordan EG, Monahan FJ, Caffrey PJ, et al Fatty acid composition, including conjugated linoleic acid, of intramuscular fat from steers offered grazed grass, grass silage, or concentrate-based diets. J Anim Sci. 2000;78(11):2849–55. 10.2527/2000.78112849x 11063308

[4] Daley CA, Abbott A, Doyle PS, Nader GA, Larson S. A review of fatty acid profiles and antioxidant content in grass-fed and grain-fed beef. Nutr J. 2010;9(10):1–12. 20219103 10.1186/1475-2891-9-10 PMC2846864

[5] USDA. USDA Agricultural Marketing Service Grass Fed Marketing Claim Standard. USDA; 2016.

[6] USDA Nutrient Database for Standard Reference, (1999).

[7] Bronkema SM, Rowntree JE, Jain R, Schweihofer JP, Bitler CA, Fenton JI. A Nutritional Survey of Commercially Available Grass-Finished Beef. Meat and Muscle Biology. 2019;3:116.

[8] Patterson E, Wall R, Fitzgerald GF, Ross RP, Stanton C. Health implications of high dietary omega-6 polyunsaturated Fatty acids. Journal of nutrition and metabolism. 2012;2012:539426 10.1155/2012/539426 22570770 PMC3335257

[9] Locke A, Schneiderhan J, Zick SM. Diets for Health: Goals and Guidelines. Am Fam Physician. 2018;97:721–8. 30215930

[10] Ponnampalam EN, Hopkins DL, Jacobs JL. Increasing omega-3 levels in meat from ruminants under pasture-based systems. Revue scientifique et technique. 2018;37(1):57–70. 10.20506/rst.37.1.2740 30209429

[11] Uzhova I, Peñalvo JL. Mediterranean diet and cardio-metabolic health: what is the role of meat? Eur J Clin Nutr. 2018. 10.1038/s41430-018-0303-y 30487568

[12] Pestana JM, Costa ASH, Alves SP, Martins SV, Alfaia CM, Bessa RJB, et al Seasonal changes and muscle type effect on the nutritional quality of intramuscular fat in Mirandesa-PDO veal. Meat science. 2012;90:819–27. 10.1016/j.meatsci.2011.11.023 22133588

[13] Sobczuk-Szul M, Wroński M, Wielgosz-Groth Z, Mochol M, Rzemieniewski A, Nogalski Z, et al The effect of slaughter season on the fatty acid profile in four types of fat deposits in crossbred beef bulls. Asian-Australasian Journal of Animal Sciences. 2013;26:275–81. 10.5713/ajas.2012.12371 25049787 PMC4093157

[14] Fernandez ML, West KL. Mechanisms by which Dietary Fatty Acids Modulate Plasma Lipids. The Journal of Nutrition. 2005;135:2075–8. 10.1093/jn/135.9.2075 16140878

[15] Martin SS, Blumenthal RS, Miller M. LDL Cholesterol: The Lower the Better. 2012;96:13–26. 10.1016/j.mcna.2012.01.009 22391248

[16] Sales-Campos H, Souza PRd, Peghini BC, da Silva JS, Cardoso CR. An overview of the modulatory effects of oleic acid in health and disease. Mini Rev Med Chem. 2013;13:201–10. 23278117

[17] Provenza FD, Kronberg SL, Gregorini P. Is Grassfed Meat and Dairy Better for Human and Environmental Health? Frontiers in nutrition. 2019;6:26 10.3389/fnut.2019.00026 30941351 PMC6434678

[18] Wood JD, Richardson RI, Scollan ND, Hopkins A, Dunn RM, Buller H, et al, editors. Quality of meat from biodiverse grassland. British Grassland Society Occasion Symposium; 2007.

[19] Dierking RM, Kallenbach RL, Roberts CA. Fatty Acid Profiles of Orchardgrass, Tall Fescue, Perennial Ryegrass, and Alfalfa. Crop Science. 2010;50:391.

[20] Scollan N, Hocquette J-F, Nuernberg K, Dannenberger D, Richardson I, Moloney A. Innovations in beef production systems that enhance the nutritional and health value of beef lipids and their relationship with meat quality. Meat science. 2006;74:17–33. 10.1016/j.meatsci.2006.05.002 22062713

[21] Scollan N, Costa P, Hallett K, Nute G, Wood J, Richardson R. The fatty acid composition of muscle fat and relationship to meat quality in Charolais steers: influence of level of red clover in the diet. BSAS; 2006 p. 23.

[22] Dewhurst RJ, Shingfield KJ, Lee MRF, Scollan ND. Increasing the concentrations of beneficial polyunsaturated fatty acids in milk produced by dairy cows in high-forage systems. Animal Feed Science and Technology. 2006;131:168–206.

[23] Fuke G, Nornberg JL. Systematic evaluation on the effectiveness of conjugated linoleic acid in human health. Crit Rev Food Sci Nutr. 2017;57:1–7. 10.1080/10408398.2012.716800 27636835

[24] Whigham LD, Watras AC, Schoeller DA. Efficacy of conjugated linoleic acid for reducing fat mass: A meta-analysis in humans. American Journal of Clinical Nutrition. 2007;85:1203–11. 10.1093/ajcn/85.5.1203 17490954

[25] Silva-Ramirez AS, Castillo CG, Navarro-Tovar G, Gonzalez-Sanchez HM, Rocha-Uribe A, Gonzalez-Chavez MM, et al Bioactive Isomers of Conjugated Linoleic Acid Inhibit the Survival of Malignant Glioblastoma Cells But Not Primary Astrocytes. European Journal of Lipid Science and Technology. 2018;120(11):1700454.

[26] Kilcher MR. Plant development, stage of maturity and nutrient composition. Journal of Range Management. 1981;34(5):363–4.

[27] Ballet N, Robert J. C., Williams P. E. V. Vitamins in Forages. Wallingford, UK: CABI Pub; 2000.

[28] Dumont B, Garel JP, Ginane C, Decuq F, Farruggia A, Pradel P, et al Effect of cattle grazing a species-rich mountain pasture under different stocking rates on the dynamics of diet selection and sward structure. Animal: an international journal of animal bioscience. 2007;1:1042–52. 22444807 10.1017/S1751731107000250

[29] Umberger WJ, Boxall PC, Lacy RC. Role of credence and health information in determining US consumers' willingness-to-pay for grass-finished beef. Aust J Agr Resour Ec. 2009;53(4):603–23.

[30] Simopoulos AP. The importance of the ratio of omega-6/omega-3 essential fatty acids. Biomedicine & pharmacotherapy. 2002;56:365–79. 12442909 10.1016/s0753-3322(02)00253-6

[31] Husted KS, Bouzinova EV. The importance of n-6/n-3 fatty acids ratio in the major depressive disorder Medicina (Lithuania): Elsevier; 2016 p. 139–47. 10.1016/j.medici.2016.05.003 27496183

[32] Leheska JM, Thompson LD, Howe JC, Hentges E, Boyce J, Brooks JC, et al Effects of conventional and grass-feeding systems on the nutrient composition of beef. J Anim Sci. 2008;86(12):3575–85. 10.2527/jas.2007-0565 18641180

[33] Alfaia CPM, Alves SP, Martins SIV, Costa ASH, Fontes CMGA, Lemos JPC, et al Effect of the feeding system on intramuscular fatty acids and conjugated linoleic acid isomers of beef cattle, with emphasis on their nutritional value and discriminatory ability. Food Chem. 2009;114:939–46.

[34] Insani EM, Sancho AM, García PT, Biolatto A, Descalzo AM, Josifovich JA, et al Influence of pasture or grain-based diets supplemented with vitamin E on antioxidant/oxidative balance of Argentine beef. Meat science. 2005;70:35–44. 10.1016/j.meatsci.2004.11.018 22063278

[35] Jain R, Austin Pickens C, Fenton JI. The role of the lipidome in obesity-mediated colon cancer risk. J Nutr Biochem. 2018;59:1–9. 10.1016/j.jnutbio.2018.02.015 29605789

[36] Wang X, Lin H, Gu Y. Multiple roles of dihomo-γ-linolenic acid against proliferation diseases. Lipids Health Dis: BioMed Central; 2012 p. 25. 10.1186/1476-511X-11-25 PMC3295719 22333072

[37] Gómez Candela C, Bermejo López LM, Loria Kohen V. Importance of a balanced omega 6/omega 3 ratio for the maintenance of health: nutritional recommendations. Nutr Hosp. 2011;26:323–9. 10.1590/S0212-16112011000200013 21666970

[38] Yang A, Lanari MC, Brewster M, Tume RK. Lipid stability and meat colour of beef from pasture- and grain-fed cattle with or without vitamin E supplement. Meat science. 2002;60:41–50. 10.1016/s0309-1740(01)00103-6 22063104

[39] Palmquist DL, Lock AL, Shingfield KJ, Bauman DE. Biosynthesis of Conjugated Linoleic Acid in Ruminants and Humans. Adv Food Nutr Res 2005 p. 179–217. 10.1016/S1043-4526(05)50006-8 16263431

[40] Turner KE, Foster JG, Borton RJ, McClure KE, Weiss WP. Alpha-tocopherol concentrations and case life of lamb muscle as influenced by concentrate or pasture finishing. J Anim Sci. 2002;80:2513–21. 12413072

[41] Insani EM, Eyherabide A, Grigioni G, Sancho AM, Pensel NA, Descalzo AM. Oxidative stability and its relationship with natural antioxidants during refrigerated retail display of beef produced in Argentina. Meat science. 2008;79:444–52. 10.1016/j.meatsci.2007.10.017 22062904

[42] Mirzad AN, Tada T, Ano H, Kobayashi I, Yamauchi T, Katamoto H. Seasonal changes in serum oxidative stress biomarkers in dairy and beef cows in a daytime grazing system. J Vet Med Sci. 2018;80(1):20–7. 10.1292/jvms.17-0321 29142148 PMC5797854

[43] Tinggi U. Selenium: Its role as antioxidant in human health: BioMed Central; 2008. 10.1007/s12199-007-0019-4 PMC2698273 19568888

[44] Akil M, Gurbuz U, Bicer M, Halifeoglu I, Baltaci AK, Mogulkoc R. Selenium prevents lipid peroxidation in liver and lung tissues of rats in acute swimming exercise. Bratisl Lek Listy. 2015;116:233–5. 10.4149/bll_2015_045 25773950

[45] Schmidt JR, Miller MC, Andrae JG, Ellis SE, Duckett SK. Effect of summer forage species grazed during finishing on animal performance, carcass quality, and meat quality. J Anim Sci. 2013;91(9):4451–61. 10.2527/jas.2012-5405 23825343

[46] Lewis RM, Duckett SK, Fontenot JP, Clapham WM, Neel JPS. Effects of forage species or concentrate finishing on animal performance, carcass and meat quality. J Anim Sci. 2013;91:1454–67. 10.2527/jas.2012-5914 23345568

[47] Preston RL. Typical composition of feeds for cattle and sheep. Beef. 1999:1–15.

[48] Yoshihara Y, Sato S. Seasonal change and distribution of grass nutritive values and minerals in an open pasture surrounded by forest. Agroforestry Systems. 2013;87:901–7.

[49] Roberts AHC. Seasonal variation in soil tests and nutrient content of pasture at two sites in taranaki. New Zealand Journal of Experimental Agriculture. 1987;15:283–94.

[50] Domaradzki P, Florek M, Staszowska A, Litwińczuk Z. Evaluation of the Mineral Concentration in Beef from Polish Native Cattle. Biol Trace Elem Res. 2016;171:328–32. 10.1007/s12011-015-0549-3 26498196 PMC4856721

[51] Tume RK. The effects of environmental factors on fatty acid composition and the assessment of marbling in beef cattle: a review. Australian Journal of Experimental Agriculture. 2004;44:663–8.

[52] Bradford HL, Fragomeni BO, Bertrand JK, Lourenco DAL, Misztal I. Regional and seasonal analyses of weights in growing angus cattle. J Anim Sci. 2016;94:4369–70. 10.2527/jas.2016-0683 27898859

[53] Steiner JL, Schneider JM, Pope C, Pope S, Ford P, Steele RF. Southern Plains Assessment of Vulnerability and Preliminary Adaptation and Mitigation Strategies for Farmers, Ranchers, and Forest Land Owners. USDA; 2015.

[54] Baumgard LH, Rhoads RP. Effects of Heat Stress on Postabsorptive Metabolism and Energetics. Annu Rev Anim Biosci. 2012(1):311–37. 25387022 10.1146/annurev-animal-031412-103644

