# Supplementary material for: Seasonal differences exist in the polyunsaturated fatty acid, mineral and antioxidant content of U.S. grass-finished beef
Source: PLoS One. 2020 Feb 26;15(2):e0229340. doi: 10.1371/journal.pone.0229340 (PMC7043805; doi:10.1371/journal.pone.0229340)
Supplement: S2 Appendix — The R code used for statistical analysis as an .html file. (HTML) [file pone.0229340.s002.html]

Seasonal differences exist in the polyunsaturated fatty acid, mineral and antioxidant content of U.S. grass-finished beef


# Seasonal differences exist in the polyunsaturated fatty acid, mineral and antioxidant content of U.S. grass-finished beef

#### 11/20/2019

###Load Data This is the cleaned code for our final analysis of grass fed beef fatty acid, mineral, and antioxidant content between seasons and producers. It is provided as a supplement to support our manuscript.

```
beef.dat=read.csv("Beef FA and minerals.csv")
X=beef.dat
X$PRODUCER<-as.factor(X$PRODUCER)
X[X==0]<-NA
table(is.na(X$Beta.Carotene), X$PRODUCER)
```

```
##        
##           2   4   5   8
##   FALSE   5  21   6 181
##   TRUE   70  85  75  69
```

```
#All used packages, I also listed them right above the code applying to them to match function and package.
if(!require(ggpubr)){install.packages("ggpubr")}
```

```
## Loading required package: ggpubr
```

```
## Loading required package: ggplot2
```

```
## Loading required package: magrittr
```

```
if(!require(agricolae)){install.packages("agricolae")}
```

```
## Loading required package: agricolae
```

Going forward, I will work through the dataframe X, a copy of beef.dat for simplicity. Replaced all 0 with NA so they are excluded from analyses testing significance.

###Normality According to the central limit theorem, parametric tests for samples sizes greater than 30 can be used as normality is assumed (population, not sample). Practically, it makes sense to run normality tests to verify the data is indeed normal.

The Shapiro test may be used where p<0.05 indicates non-normal distribution. This can be further visualized through histograme or Q-Q plots. Note that the last plot is of log transformed data which normalizes the data allowing for the use of parametric tests for significant differences such as t tests and ANOVAs. Further, please note that the Welch t-test and Unbalanced ANOVA will be used as sample sizes are different between the various groups (producers, seasons, etc.)

```
shapiro.test(X$Total.FA)
```

```
## 
##  Shapiro-Wilk normality test
## 
## data:  X$Total.FA
## W = 0.86633, p-value < 2.2e-16
```

```
hist(X$Total.FA, breaks=40, main = "Total FA", xlab = "")
```

```
require(ggpubr)
ggqqplot(X$Total.FA, title = "Total FA")
```

```
ggqqplot(X[X$Season.x=="Fall",]$Total.FA, title = "Total Fall FA")
```

```
ggqqplot(X[X$Season.x=="Spring",]$Total.FA, title = "Total Spring FA")
```

```
ggqqplot(X[X$PRODUCER=="8" & X$Season.x=="Spring",]$Total.FA, title = "P2 Total Spring FA")
```

```
ggqqplot(X[X$PRODUCER=="8" & X$Season.x=="Fall",]$Total.FA, title = "P2 Total Fall FA")
```

```
ggqqplot(log10(X$Total.FA), title = "Log10(Total FA)")
```

###Tables 1 and 2 data Below is info for Table 1 and 2.

```
table(X$PRODUCER,X$Season.x)
```

```
##    
##     Fall Spring
##   2   25     50
##   4   35     71
##   5   43     38
##   8   99    151
```

```
Z=matrix(NA, nrow = 4, ncol = 5)
colnames(Z)=c("P2","P4","P5","P8", "prod.p")
rownames(Z)=c("Total FA", "Fall FA", "Spring FA", "Season.p")

#Total FA

Z[1,1]=paste0(round(mean(X[X$PRODUCER=="2",]$Total.FA, na.rm = T),1),"±",round((sd(X[X$PRODUCER=="2",]$Total.FA,na.rm = T)/(sqrt(nrow(X[X$PRODUCER=="2",])))),1))
Z[1,2]=paste0(round(mean(X[X$PRODUCER=="4",]$Total.FA, na.rm = T),1),"±",round((sd(X[X$PRODUCER=="4",]$Total.FA,na.rm = T)/(sqrt(nrow(X[X$PRODUCER=="4",])))),1))
Z[1,3]=paste0(round(mean(X[X$PRODUCER=="5",]$Total.FA, na.rm = T),1),"±",round((sd(X[X$PRODUCER=="5",]$Total.FA,na.rm = T)/(sqrt(nrow(X[X$PRODUCER=="5",])))),1))
Z[1,4]=paste0(round(mean(X[X$PRODUCER=="8",]$Total.FA, na.rm = T),1),"±",round((sd(X[X$PRODUCER=="8",]$Total.FA,na.rm = T)/(sqrt(nrow(X[X$PRODUCER=="8",])))),1))
Z[1,5]=round(summary(aov(log10(X$Total.FA)~X$PRODUCER))[[1]][1,5],3)

#Fall FA
Z[2,1]=paste0(round(mean(X[X$PRODUCER=="2" & X$Season.x=="Fall",]$Total.FA, na.rm = T),1),"±",round((sd(X[X$PRODUCER=="2" & X$Season.x=="Fall",]$Total.FA,na.rm = T)/(sqrt(nrow(X[X$PRODUCER=="2" & X$Season.x=="Fall",])))),1))
Z[2,2]=paste0(round(mean(X[X$PRODUCER=="4" & X$Season.x=="Fall",]$Total.FA, na.rm = T),1),"±",round((sd(X[X$PRODUCER=="4" & X$Season.x=="Fall",]$Total.FA,na.rm = T)/(sqrt(nrow(X[X$PRODUCER=="4" & X$Season.x=="Fall",])))),1))
Z[2,3]=paste0(round(mean(X[X$PRODUCER=="5" & X$Season.x=="Fall",]$Total.FA, na.rm = T),1),"±",round((sd(X[X$PRODUCER=="5" & X$Season.x=="Fall",]$Total.FA,na.rm = T)/(sqrt(nrow(X[X$PRODUCER=="5" & X$Season.x=="Fall",])))),1))
Z[2,4]=paste0(round(mean(X[X$PRODUCER=="8" & X$Season.x=="Fall",]$Total.FA, na.rm = T),1),"±",round((sd(X[X$PRODUCER=="8" & X$Season.x=="Fall",]$Total.FA,na.rm = T)/(sqrt(nrow(X[X$PRODUCER=="8" & X$Season.x=="Fall",])))),1))
Z[2,5]=round(summary(aov(log10(X[X$Season.x=="Fall",]$Total.FA)~X[X$Season.x=="Fall",]$PRODUCER))[[1]][1,5],3)

#Spring FA
Z[3,1]=paste0(round(mean(X[X$PRODUCER=="2" & X$Season.x=="Spring",]$Total.FA, na.rm = T),1),"±",round((sd(X[X$PRODUCER=="2" & X$Season.x=="Spring",]$Total.FA,na.rm = T)/(sqrt(nrow(X[X$PRODUCER=="2" & X$Season.x=="Spring",])))),1))
Z[3,2]=paste0(round(mean(X[X$PRODUCER=="4" & X$Season.x=="Spring",]$Total.FA, na.rm = T),1),"±",round((sd(X[X$PRODUCER=="4" & X$Season.x=="Spring",]$Total.FA,na.rm = T)/(sqrt(nrow(X[X$PRODUCER=="4" & X$Season.x=="Spring",])))),1))
Z[3,3]=paste0(round(mean(X[X$PRODUCER=="5" & X$Season.x=="Spring",]$Total.FA, na.rm = T),1),"±",round((sd(X[X$PRODUCER=="5" & X$Season.x=="Spring",]$Total.FA,na.rm = T)/(sqrt(nrow(X[X$PRODUCER=="5" & X$Season.x=="Spring",])))),1))
Z[3,4]=paste0(round(mean(X[X$PRODUCER=="8" & X$Season.x=="Spring",]$Total.FA, na.rm = T),1),"±",round((sd(X[X$PRODUCER=="8" & X$Season.x=="Spring",]$Total.FA,na.rm = T)/(sqrt(nrow(X[X$PRODUCER=="8" & X$Season.x=="Spring",])))),1))
Z[3,5]=round(summary(aov(log10(X[X$Season.x=="Spring",]$Total.FA)~X[X$Season.x=="Spring",]$PRODUCER))[[1]][1,5],3)

#Intraproducer seasonal p-values
Z[4,1]=round(t.test(log10(X[X$PRODUCER=="2" & X$Season.x=="Spring",]$Total.FA),log10(X[X$PRODUCER=="2" & X$Season.x=="Fall",]$Total.FA))$p.val,3)
Z[4,2]=round(t.test(log10(X[X$PRODUCER=="4" & X$Season.x=="Spring",]$Total.FA),log10(X[X$PRODUCER=="4" & X$Season.x=="Fall",]$Total.FA))$p.val,3)
Z[4,3]=round(t.test(log10(X[X$PRODUCER=="5" & X$Season.x=="Spring",]$Total.FA),log10(X[X$PRODUCER=="5" & X$Season.x=="Fall",]$Total.FA))$p.val,3)
Z[4,4]=round(t.test(log10(X[X$PRODUCER=="8" & X$Season.x=="Spring",]$Total.FA),log10(X[X$PRODUCER=="8" & X$Season.x=="Fall",]$Total.FA))$p.val,3)

print(Z)
```

```
##           P2             P4            P5           P8           prod.p
## Total FA  "935±60"       "1121.8±54"   "834.5±59.4" "544.2±17.2" "0"   
## Fall FA   "1249.6±123.1" "822.6±68.4"  "828.2±83.7" "538.9±27"   "0"   
## Spring FA "777.7±54.2"   "1269.3±66.9" "841.6±85.2" "547.7±22.3" "0"   
## Season.p  "0.002"        "0"           "0.828"      "0.904"      NA
```

```
write.csv(Z, "Table_2.csv")

#Tukey's pairwise comparison for producers; input results by hand

TukeyHSD(aov(log10(X$Total.FA)~X$PRODUCER), ordered = TRUE)
```

```
##   Tukey multiple comparisons of means
##     95% family-wise confidence level
##     factor levels have been ordered
## 
## Fit: aov(formula = log10(X$Total.FA) ~ X$PRODUCER)
## 
## $`X$PRODUCER`
##           diff          lwr       upr     p adj
## 5-8 0.15261184  0.078015680 0.2272080 0.0000012
## 2-8 0.22098245  0.144165688 0.2977992 0.0000000
## 4-8 0.31569190  0.248065377 0.3833184 0.0000000
## 2-5 0.06837062 -0.025127774 0.1618690 0.2358270
## 4-5 0.16308006  0.076972687 0.2491874 0.0000084
## 4-2 0.09470944  0.006671336 0.1827476 0.0293229
```

```
TukeyHSD(aov(log10(X[X$Season.x=="Fall",]$Total.FA)~X[X$Season.x=="Fall",]$PRODUCER), ordered = TRUE)
```

```
##   Tukey multiple comparisons of means
##     95% family-wise confidence level
##     factor levels have been ordered
## 
## Fit: aov(formula = log10(X[X$Season.x == "Fall", ]$Total.FA) ~ X[X$Season.x == "Fall", ]$PRODUCER)
## 
## $`X[X$Season.x == "Fall", ]$PRODUCER`
##           diff         lwr       upr     p adj
## 5-8 0.14825370  0.04079592 0.2557115 0.0024692
## 4-8 0.18895373  0.07325029 0.3046572 0.0002068
## 2-8 0.35394639  0.22225156 0.4856412 0.0000000
## 4-5 0.04070004 -0.09324436 0.1746444 0.8601684
## 2-5 0.20569269  0.05771518 0.3536702 0.0022464
## 2-4 0.16499266  0.01092301 0.3190623 0.0305790
```

```
TukeyHSD(aov(log10(X[X$Season.x=="Spring",]$Total.FA)~X[X$Season.x=="Spring",]$PRODUCER), ordered = TRUE)
```

```
##   Tukey multiple comparisons of means
##     95% family-wise confidence level
##     factor levels have been ordered
## 
## Fit: aov(formula = log10(X[X$Season.x == "Spring", ]$Total.FA) ~ X[X$Season.x == "Spring", ]$PRODUCER)
## 
## $`X[X$Season.x == "Spring", ]$PRODUCER`
##            diff         lwr       upr     p adj
## 2-8 0.154193732  0.06305702 0.2453304 0.0000998
## 5-8 0.158481539  0.05710921 0.2598539 0.0003945
## 4-8 0.377847821  0.29747159 0.4582241 0.0000000
## 5-2 0.004287807 -0.11592018 0.1244958 0.9997192
## 4-2 0.223654090  0.12053305 0.3267751 0.0000003
## 4-5 0.219366282  0.10709697 0.3316356 0.0000046
```

###Tables 3 and 4 data Calculate mean and SEM for all data. This is info going into Tables 3 and 4.

```
Z=matrix(NA, nrow = ncol(X), nc=6)
colnames(Z)=c("Overall","Fall","Spring", "Season.p", "Producer.p", "Interaction.p")
rownames(Z)=colnames(X)
for (i in 4:ncol(X)){
  Z[i,1]=paste0(round(mean(X[,i],na.rm = TRUE),2),"±",round((sd(X[,i],na.rm = TRUE))/(sqrt(nrow(X))),2)) 
  Z[i,2]=paste0(round(mean(X[X$Season.x=="Fall",i],na.rm=TRUE),2),"±",round((sd(X[X$Season.x=="Fall",i],na.rm=TRUE))/(sqrt(nrow(X[X$Season.x=="Fall",]))),2)) 
  Z[i,3]=paste0(round(mean(X[X$Season.x=="Spring",i],na.rm=TRUE),2),"±",round((sd(X[X$Season.x=="Spring",i],na.rm=TRUE))/(sqrt(nrow(X[X$Season.x=="Spring",]))),2))
  Z[i,4]=round(summary(aov(log10(X[,i])~X$Season.x*X$PRODUCER))[[1]][1,5],3)
  Z[i,5]=round(summary(aov(log10(X[,i])~X$Season.x*X$PRODUCER))[[1]][2,5],3)
  Z[i,6]=round(summary(aov(log10(X[,i])~X$Season.x*X$PRODUCER))[[1]][3,5],3)
}

#Preview of data
head(Z)
```

```
##            Overall        Fall           Spring         Season.p Producer.p
## Working.ID NA             NA             NA             NA       NA        
## PRODUCER   NA             NA             NA             NA       NA        
## Season.x   NA             NA             NA             NA       NA        
## SFA        "340.45±10.02" "331.2±16"     "346.47±12.86" "0.355"  "0"       
## MUFA       "340.74±10.76" "327.12±16.79" "349.62±14"    "0.229"  "0"       
## PUFA       "85.76±1.43"   "79.29±1.83"   "89.98±2.01"   "0"      "0"       
##            Interaction.p
## Working.ID NA           
## PRODUCER   NA           
## Season.x   NA           
## SFA        "0"          
## MUFA       "0"          
## PUFA       "0.001"
```

```
write.csv(Z, file = "Table3_4.csv")
```

###Table 5 and 6 data Generate all data for tables 5 ands 6. Only n-3 and n-6 data will be in the manuscript table but all other FA data will be available in a supplement

```
Z=matrix(NA, nrow = ncol(X), nc=12)
colnames(Z)=c("P2 F","P2 S","P2.season.p","P4 F","P4 S","P4.season.p","P5 F","P5 S","P5.season.p","P8 F","P8 S","P8.season.p")
rownames(Z)=colnames(X)
for (i in 4:(ncol(X)-2)){
  Z[i,1]=paste0(round(mean(X[X$PRODUCER=="2" & X$Season.x=="Fall",i],na.rm=TRUE),2),"±",round((sd(X[X$PRODUCER=="2" & X$Season.x=="Fall",i],na.rm=TRUE))/(sqrt(nrow(X[X$PRODUCER=="2" & X$Season.x=="Fall",]))),2)) 
  Z[i,2]=paste0(round(mean(X[X$PRODUCER=="2" & X$Season.x=="Spring",i],na.rm=TRUE),2),"±",round((sd(X[X$PRODUCER=="2" & X$Season.x=="Spring",i],na.rm=TRUE))/(sqrt(nrow(X[X$PRODUCER=="2" & X$Season.x=="Spring",]))),2)) 
  Z[i,3]=round(t.test(log10(X[X$PRODUCER=="2" & X$Season.x=="Spring",i]), log10(X[X$PRODUCER=="2" & X$Season.x=="Fall",i]))$p.val,3)

  Z[i,4]=paste0(round(mean(X[X$PRODUCER=="4" & X$Season.x=="Fall",i],na.rm=TRUE),2),"±",round((sd(X[X$PRODUCER=="4" & X$Season.x=="Fall",i],na.rm=TRUE))/(sqrt(nrow(X[X$PRODUCER=="4" & X$Season.x=="Fall",]))),2)) 
  Z[i,5]=paste0(round(mean(X[X$PRODUCER=="4" & X$Season.x=="Spring",i],na.rm=TRUE),2),"±",round((sd(X[X$PRODUCER=="4" & X$Season.x=="Spring",i],na.rm=TRUE))/(sqrt(nrow(X[X$PRODUCER=="4" & X$Season.x=="Spring",]))),2)) 
  Z[i,6]=round(t.test(log10(X[X$PRODUCER=="4" & X$Season.x=="Spring",i]), log10(X[X$PRODUCER=="4" & X$Season.x=="Fall",i]))$p.val,3)
  
  Z[i,7]=paste0(round(mean(X[X$PRODUCER=="5" & X$Season.x=="Fall",i],na.rm=TRUE),2),"±",round((sd(X[X$PRODUCER=="5" & X$Season.x=="Fall",i],na.rm=TRUE))/(sqrt(nrow(X[X$PRODUCER=="5" & X$Season.x=="Fall",]))),2))  
  Z[i,8]=paste0(round(mean(X[X$PRODUCER=="5" & X$Season.x=="Spring",i],na.rm=TRUE),2),"±",round((sd(X[X$PRODUCER=="5" & X$Season.x=="Spring",i],na.rm=TRUE))/(sqrt(nrow(X[X$PRODUCER=="5" & X$Season.x=="Spring",]))),2))  
  Z[i,9]=round(t.test(log10(X[X$PRODUCER=="5" & X$Season.x=="Spring",i]), log10(X[X$PRODUCER=="5" & X$Season.x=="Fall",i]))$p.val,3)
  
  Z[i,10]=paste0(round(mean(X[X$PRODUCER=="8" & X$Season.x=="Fall",i],na.rm=TRUE),2),"±",round((sd(X[X$PRODUCER=="8" & X$Season.x=="Fall",i],na.rm=TRUE))/(sqrt(nrow(X[X$PRODUCER=="8" & X$Season.x=="Fall",]))),2)) 
  Z[i,11]=paste0(round(mean(X[X$PRODUCER=="8" & X$Season.x=="Spring",i],na.rm=TRUE),2),"±",round((sd(X[X$PRODUCER=="8" & X$Season.x=="Spring",i],na.rm=TRUE))/(sqrt(nrow(X[X$PRODUCER=="8" & X$Season.x=="Spring",]))),2)) 
  Z[i,12]=round(t.test(log10(X[X$PRODUCER=="8" & X$Season.x=="Spring",i]), log10(X[X$PRODUCER=="8" & X$Season.x=="Fall",i]))$p.val,3)
}

#Preview
head(Z)
```

```
##            P2 F          P2 S           P2.season.p P4 F          
## Working.ID NA            NA             NA          NA            
## PRODUCER   NA            NA             NA          NA            
## Season.x   NA            NA             NA          NA            
## SFA        "575.7±62.01" "350.22±25.24" "0.002"     "375.13±33.13"
## MUFA       "597.3±60.4"  "357.43±28.29" "0.002"     "345.3±32.82" 
## PUFA       "76.6±4.35"   "69.99±2.35"   "0.243"     "102.16±4.75" 
##            P4 S           P4.season.p P5 F           P5 S           P5.season.p
## Working.ID NA             NA          NA             NA             NA         
## PRODUCER   NA             NA          NA             NA             NA         
## Season.x   NA             NA          NA             NA             NA         
## SFA        "564.51±30.69" "0"         "367.83±38.69" "368.86±37.27" "0.867"    
## MUFA       "570.14±33.87" "0"         "372.02±43.48" "377.33±45.18" "0.831"    
## PUFA       "134.61±4.22"  "0"         "88.32±4.07"   "95.44±4.54"   "0.236"    
##            P8 F           P8 S           P8.season.p
## Working.ID NA             NA             NA         
## PRODUCER   NA             NA             NA         
## Season.x   NA             NA             NA         
## SFA        "238.02±12.82" "237.08±10.4"  "0.834"    
## MUFA       "232.96±13.47" "236.37±11.19" "0.915"    
## PUFA       "67.96±1.84"   "74.24±1.56"   "0.007"
```

```
write.csv(Z, file = "Table5_6.csv")

#Calculate beta-carotene values and season t-test for Prod 4 and 8

#Prod 2 Fall
paste0(round(mean(X[X$PRODUCER=="2" & X$Season.x=="Fall",]$Beta.Carotene,na.rm=TRUE),2),"±",round((sd(X[X$PRODUCER=="2" & X$Season.x=="Fall",]$Beta.Carotene,na.rm=TRUE))/(sqrt(nrow(X[X$PRODUCER=="2" & X$Season.x=="Fall",]))),2))
```

```
## [1] "37.4±1.64"
```

```
#Prod 4 Fall and Spring
paste0(round(mean(X[X$PRODUCER=="4" & X$Season.x=="Fall",]$Beta.Carotene,na.rm=TRUE),2),"±",round((sd(X[X$PRODUCER=="4" & X$Season.x=="Fall",]$Beta.Carotene,na.rm=TRUE))/(sqrt(nrow(X[X$PRODUCER=="4" & X$Season.x=="Fall",]))),2))
```

```
## [1] "26.4±0.54"
```

```
paste0(round(mean(X[X$PRODUCER=="4" & X$Season.x=="Spring",]$Beta.Carotene,na.rm=TRUE),2),"±",round((sd(X[X$PRODUCER=="4" & X$Season.x=="Spring",]$Beta.Carotene,na.rm=TRUE))/(sqrt(nrow(X[X$PRODUCER=="4" & X$Season.x=="Spring",]))),2))
```

```
## [1] "32.27±2.81"
```

```
round(t.test(log10(X[X$PRODUCER=="4" & X$Season.x=="Spring",]$Beta.Carotene), log10(X[X$PRODUCER=="4" & X$Season.x=="Fall",]$Beta.Carotene))$p.val,3)
```

```
## [1] 0.567
```

```
#Prod 5 Fall
paste0(round(mean(X[X$PRODUCER=="5" & X$Season.x=="Fall",]$Beta.Carotene,na.rm=TRUE),2),"±",round((sd(X[X$PRODUCER=="5" & X$Season.x=="Fall",]$Beta.Carotene,na.rm=TRUE))/(sqrt(nrow(X[X$PRODUCER=="5" & X$Season.x=="Fall",]))),2))
```

```
## [1] "30.33±1.23"
```

```
#Prod 8 Fall and Spring
paste0(round(mean(X[X$PRODUCER=="8" & X$Season.x=="Fall",]$Beta.Carotene,na.rm=TRUE),2),"±",round((sd(X[X$PRODUCER=="8" & X$Season.x=="Fall",]$Beta.Carotene,na.rm=TRUE))/(sqrt(nrow(X[X$PRODUCER=="8" & X$Season.x=="Fall",]))),2))
```

```
## [1] "34.4±1.12"
```

```
paste0(round(mean(X[X$PRODUCER=="8" & X$Season.x=="Spring",]$Beta.Carotene,na.rm=TRUE),2),"±",round((sd(X[X$PRODUCER=="8" & X$Season.x=="Spring",]$Beta.Carotene,na.rm=TRUE))/(sqrt(nrow(X[X$PRODUCER=="8" & X$Season.x=="Spring",]))),2))
```

```
## [1] "30.08±0.56"
```

```
round(t.test(log10(X[X$PRODUCER=="8" & X$Season.x=="Spring",]$Beta.Carotene), log10(X[X$PRODUCER=="8" & X$Season.x=="Fall",]$Beta.Carotene))$p.val,3)
```

```
## [1] 0.019
```

```
#HSD results for Fall
Z=matrix(NA, ncol = 4)
colnames(Z)<-c("2","4","5","8")
X1=X[X$Season.x=="Fall",]
require(agricolae)
for (i in 4:(ncol(X1)-2)){
 myanova=aov(log10(X1[,i])~X1$PRODUCER)
 Y=HSD.test(myanova,"X1$PRODUCER", group = T)
 a<-t(Y$groups[2])
 Z<-rbind(Z,a[,colnames(Z)])
} 
Z1<-cbind(Z[2:nrow(Z),],colnames(X1)[4:(ncol(X1)-2)])
colnames(Z1)[5]<-"Variable"

#Preview
head(Z1)
```

```
##      2    4   5    8   Variable 
## [1,] "a"  "b" "b"  "c" "SFA"    
## [2,] "a"  "b" "b"  "c" "MUFA"   
## [3,] "bc" "a" "ab" "c" "PUFA"   
## [4,] "c"  "a" "b"  "c" "n.6"    
## [5,] "a"  "b" "b"  "a" "n.3"    
## [6,] "c"  "a" "b"  "c" "n.6.n.3"
```

```
write.csv(Z1,file = "Fall_prod_Tukey.csv")

#HSD results for spring
Z=matrix(NA, ncol = 4)
colnames(Z)<-c("2","4","5","8")
X2=X[X$Season.x=="Spring",]
require(agricolae)
for (i in 4:(ncol(X2)-2)){
 myanova=aov(log10(X2[,i])~X2$PRODUCER)
 Y=HSD.test(myanova,"X2$PRODUCER", group = T)
 a<-t(Y$groups[2])
 Z<-rbind(Z,a[,colnames(Z)])
} 
Z2<-cbind(Z[2:nrow(Z),],colnames(X2)[4:(ncol(X2)-2)])
colnames(Z2)[5]<-"Variable"

#HSD for beta carotene Fall only
myanova=aov(log10(X1$Beta.Carotene)~X1$PRODUCER)
Y=HSD.test(myanova,"X1$PRODUCER", group = T)
a<-t(Y$groups[2])

table(is.na(X$Beta.Carotene), X$PRODUCER)
```

```
##        
##           2   4   5   8
##   FALSE   5  21   6 181
##   TRUE   70  85  75  69
```

```
#Preview
head(Z2)
```

```
##      2   4   5   8   Variable 
## [1,] "b" "a" "b" "c" "SFA"    
## [2,] "b" "a" "b" "c" "MUFA"   
## [3,] "c" "a" "b" "c" "PUFA"   
## [4,] "c" "a" "b" "c" "n.6"    
## [5,] "b" "c" "c" "a" "n.3"    
## [6,] "c" "a" "b" "d" "n.6.n.3"
```

```
write.csv(Z2,file = "Spring_prod_Tukey.csv")
```

###Supplement: Median and IQR for % comp data As the data has some skew, and to make it easier for researchers to compare our data to literature reporting in other units, we are presenting median and IQR values for all FA data as % of total FAs. As we are also providing the full, quantitative dataset, median values in units mg FA/100g tissue can be independently calculated by researchers, if desired.

```
percent.comp=X[,c(2,3,18:34,4:8,11)]
X3=cbind(percent.comp[,1:2],round(100*percent.comp[,3:24]/percent.comp$Total.FA,4))

Z=matrix(NA, nrow = ncol(X3), ncol = 3)
colnames(Z)<-c("Overall","Fall","Spring")
rownames(Z)<-colnames(X3)

for (i in 3:24){
  Z[i,1]=paste0(round(median(X3[,i], na.rm = T), 3), "±" ,round(IQR(X3[,i], na.rm = T), 3))
  Z[i,2]=paste0(round(median(X3[X3$Season.x=="Fall",i], na.rm = T), 3), "±" ,round(IQR(X3[X3$Season.x=="Fall",i], na.rm = T), 3))
  Z[i,3]=paste0(round(median(X3[X3$Season.x=="Spring",i], na.rm = T), 3), "±" ,round(IQR(X3[X3$Season.x=="Spring",i], na.rm = T), 3))
}

head(Z)
```

```
##             Overall        Fall           Spring        
## PRODUCER    NA             NA             NA            
## Season.x    NA             NA             NA            
## Myristic    "1.853±0.752"  "1.917±0.728"  "1.829±0.798" 
## Myristoleic "0.512±0.267"  "0.569±0.256"  "0.478±0.278" 
## Palmitic    "27.226±3.798" "27.348±4.205" "27.203±3.445"
## Palmitoleic "3.064±1.108"  "3.035±0.966"  "3.085±1.201"
```

```
write.csv(Z, file = "S1_Table_percent.csv")
```
